# The "spare parts person"? Conceptions of the human body and their implications for public attitudes towards organ donation and organ sale

**DOI:** 10.1186/1747-5341-4-4

**Published:** 2009-02-18

**Authors:** Mark Schweda, Silke Schicktanz

**Affiliations:** 1 Department for Medical Ethics and History of Medicine, Goettingen University, Humboldtallee 36, 37073 Goettingen, Germany

## Abstract

**Background:**

The increasing debate on financial incentives for organ donation raises concerns about a "commodification of the human body". Philosophical-ethical stances on this development depend on assumptions concerning the body and how people think about it. In our qualitative empirical study we analyze public attitudes towards organ donation in their specific relation to conceptions of the human body in four European countries (Cyprus, Germany, the Netherlands and Sweden). This approach aims at a more context-sensitive picture of what "commodification of the body" can mean in concrete clinical decisions concerning organ donation.

**Results:**

We find that moral intuitions concerning organ donation are rooted in various conceptions of the human body and its relation to the self: a) the body as a mechanical object owned by the self, b) the body as a part of a higher order embodying the self, and c) the body as a hierarchy of organs constitutive of the self.

**Conclusion:**

The language of commodification is much too simple to capture what is at stake in everyday life intuitions about organ donation and organ sale. We discuss how the plurality of underlying body-self conceptions can be taken into account in the ethical debate, pointing out consequences for an anthropologically informed approach and for a liberal perspective.

## Introduction

In September 1999, visitors to the internet auction website eBay were presented with an unconventional offer: A human kidney, praised as "fully functional" in the accompanying advertisement text. Bidding for the organ began at $25,000 and soon reached $5,750,100, but since organ trafficking constitutes a criminal offence under the US-*National Organ Transplants Act*, eBay stopped the auction as soon as it was informed. Nevertheless, the case attracted broad media attention and aroused considerable public debate [1-3].

The incident could easily be dismissed as just another bizarre internet episode, and was in any case probably a hoax [4]. In two respects, however, it appears to be quite characteristic of certain tendencies in contemporary society [5]: First, it seems to represent a general trend of commercialization affecting more and more areas of personal life and social relationships, now even encroaching upon the human body and turning it into a commodity (indeed, real offers of human kidneys are still easy to find on the internet) [6]. And secondly, the crossing of this last, "physical" border obviously provokes a culturally deeply-rooted unease among the general public in most western countries.

Subsumed under the catchphrase "commodification of the human body", the critical reflection of this development constitutes an important issue in current bioethics, most prominently in the debates about market models for blood, tissue and organ procurement [7,8]. However, while the commodification debate seems to touch upon very strong intuitions about the nature of the body and its moral implications, these intuitions are often not easy to explain and to translate into rational, intersubjectively convincing bioethical arguments [9]. In the spirit of empirically-informed ethics [10], one step in this direction could be to take into account the general public and explore their views and attitudes in order to achieve a more context-sensitive picture of the positions and arguments occurring.

Against this background, we want to empirically investigate the conceptions of the human body involved in the public debate and their role in public attitudes towards organ transplantation: How are the body and its parts perceived and conceptualized and what are the implications for the evaluation of different modes of organ procurement from altruistic donation to profit-oriented sale? We start with a theoretical overview of body conceptions in recent ethical and political discussions about the commercialization of organ donation. In the methodological section, we give a short description of our own research methods. Our analysis is based on socio-empirical material from focus group discussions on transplantation medicine made up of lay people and patients in four European countries (Cyprus, Germany, the Netherlands, and Sweden) [11]. We examine three different body conceptions brought forward by the participants and their argumentative use, showing that the language of commodification is much too simple to capture what is at stake in everyday life intuitions about organ sale. In the discussion, we revisit the theoretical level in the light of our empirical findings and draw conclusions for the ethical and political debate on organ donation and its commercialization.

## Background: The human body in the debate of organ donation and its commercialization

The commodification debate shows paradigmatically that an issue like organ donation and especially organ trade [12] not only concerns our explicit evaluative and normative standards, but also culturally deeply-rooted ideas concerning human nature and existence, personhood, personal identity and the body [13,14]. The concept of commodification [15] entails that an entity is viewed and treated as a commodity, that is, an instrumental object without subjectivity and intrinsic value which can be replaced by similar objects or money [16]. Therefore, commodification arguments for (or against) the commercialization of organ procurement obviously draw on some conception of the human body which specifies why it is (or is not) adequate to view and treat it this way.

In the Kantian tradition, for example, scholars usually assume that the body is an essential part of the person as such and that persons generally have dignity, that is, incomparable value, and represent an end in themselves. Kantian philosophers therefore conclude that it would be wrong to use parts of our bodies „as a means only” [17] or even sell them because this would infringe upon our moral status as persons [18]. And especially in Marxist social philosophy, criticizing the adverse impacts of universal commodification in modern capitalistic society in terms of "commodity fetishism" and "alienation" has a long tradition. In analogy to Marx's considerations about the commodification of labor power, markets for body parts are deemed problematic because of their exploitative nature and dehumanizing effects on individuals and societies ([19], p. 3f.). On the other hand, many proponents of a commercialization of organ procurement [20] state that there is nothing wrong with commodification. Premising Locke's idea that everyone is the rightful owner of his person and faculties, especially some liberals derive a specific conception of "self-ownership" which entails that "each person is free to do with his body whatever he chooses so long as he does not cause or threaten any harm to non-consenting others" ([21], p. 40). Since most people tend to associate ownership with the right to alienation, this conception also encompasses the freedom to sell parts of one's body [22]. This line of thought seems to presuppose that the self can act as an autonomous authority disposing over its body like over some kind of property [23].

Thus, on both sides of the debate, addressing the question as to whether commodification of the body and its parts is justifiable apparently entails certain basic assumptions regarding what the body means for the self and the person as such [24]. In order to give an overview of these assumptions and systematize their role in the debate, Joralemon and Cox ([25], p. 28) have introduced a conceptual matrix. According to them, the spectrum of possible standpoints can be roughly structured along two axes: (a) a variety of *approaches to organ acquisition *resting on different degrees of voluntariness which range from altruistic donation with prior consent on the one end of the scale over several stages of external motivation by financial incentives to coercion via conscription on the other; and (b) a scale of *conceptions of the self and its relation to the body *which range from the monistic idea of the body as identical with the self on the one hand to a dualistic notion of the body as a piece of property of the detached self on the other. According to this scheme, for example, donation on the basis of a narrow consent solution is the result of a voluntary and purely altruistic (that is: supererogatory) choice and presupposes a monistic idea of the body as equatable with the self. On the other side of the spectrum are inter-vivos sale of organs. They are located in the less voluntary/more dualistic quadrant because potential donors are supposed to be coerced to do something they would not otherwise do on the basis of a body-as-property paradigm.

Clearly, these interrelations between body conceptions, conceptions of commodity and the human being are in need of further investigation – not only from a philosophical point of view, but also on the level of empirically informed ethics. Otherwise, the dynamics of individual and social decisions taking place in concrete situations cannot be adequately addressed [26].

## Methods

In the light of the academic commodification debate, our research is interested in the conceptions of the human body which actually underlie public attitudes towards organ donation in Europe. This research interest aims at a deeper understanding of public opinions by exploring their ideational and motivational backgrounds, that is, the subjective meanings they express and the cultural web of ideas and values they are embedded in. Given the specific direction of this interest and the lack of precedent research, methodological standards and systematic knowledge in this field, we used qualitative methods to gain access to this symbolic dimension.

### Composition of samples

In qualitative socio-empirical research, focus groups, moderated group discussions with usually not more than 8–10 participants [27], are an established tool for investigating common sense beliefs and public topoi on a general level [28]. Our analysis is based on the transcripts of eight focus groups which were conducted in four European countries: Cyprus, Germany, the Netherlands and Sweden.

The selection of countries was guided by an attempt to obtain a rough cross-section of the variety of national regulatory and organizational frameworks of organ transplantation in Europe. Of course the findings of qualitative studies are not representative for a country or even Europe as a whole. But an inter-European comparison allows for building hypotheses about public moralities by abstracting from specific national or religious backgrounds.

Two focus groups with 8–10 participants per group were set up in each country. One consisted of lay people and one of affected persons. The latter were patients who had had a transplant, were waiting for a transplant or had refused transplantation, along with relatives of such patients. In total, 66 European citizens took part, 34 men and 32 women. The participants were recruited using different strategies such as the distribution of flyers, online and print advertisements or the snowball method. The affected people were approached more directly with the support of medical centers, self-help groups and patients' organizations. The overall number of responses was in Germany 85, in the Netherlands 71 and in Sweden 34 (for Cyprus, no figures on overall response are available).

The composition of the groups was intended to achieve a gender balance and to be as heterogeneous as possible in regard to age and educational level. In regard to religion, however, the composition often mirrored the respective national situation; thus, in the Cypriot groups, all participants were of the Christian Orthodox faith, whereas the Swedish groups showed a dominance of Protestants. A degree of religiosity could not be given.

**Table 1:** Group composition according to socio-demographic criteria

	**CYP**		**GER**		**NED**		**SWE**	
	**aff**	**lay**	**aff**	**lay**	**aff**	**lay**	**aff**	**lay**
**Gender**	m: 6/f: 3	m: 4/f : 4	m: 5/f: 3	M: 5/f: 5	m: 3/f: 6	m: 3/f: 4	m: 4/f: 3	M: 1/f: 7
**Age**	18–30: 2; 31–45: 1; 46–60: 5; > 60: 1	18–30: 4; 31–45: 2; 46–60: 2	18–30: 1, 46–60: 3, > 60: 4	18–30: 5; 31–45: 4; 46–60: 1	18–30: 1; 31–45: 4; 46–60: 2; > 60: 2	18–30: 4; 31–45: 1; 46–60: 2	31–45: 2; 46–60: 3; > 60: 2	31–45: 1; 46–60: 1; > 60: 6
**Education**	Sec. school dipl.: 4; univ. degr.: 5	Sec. school: 2; univ. degr. 6	Voc. school/appr.: 4; sec. school dipl.: 3; without degr.: 1	Voc. school/appr.: 1; sec. school dipl.: 6; univ. degr.: 3	Voc. school/appr.: 3; sec. school dipl.: 3; univ. degr.: 3	Univ. degr. 7	Voc. school/appr.: 2; sec. school dipl.: 4 ^(inf.missing for 1 person^	Voc. school/appr.: 2; univ. degr.: 6
**Religion**	9 chr. orth.	8 chr. orth.	3 pr., 1 isl., 4 no	2 cat., 4 pr., 4 no	2 pr.; 1 ca.; 1 oth. chr. den.; 1 isl., 4 no	2 pr.; 2 cat.; 3 no	5 pr.; 2 no	6 pr.; 2 no

### Method of data collection and analysis

In all countries, the group discussions were moderated by two facilitators and followed the same semi-structured questionnaire. The questionnaire contained a) a future scenario of unlimited organ replacement, b) questions about a hypothetical case of a proxy decision for a brain-dead relative and open questions about c) attitudes towards post-mortem and living donation, and d) public policy. It was designed to initiate discussion and kick-off a discursive dynamic through which participants would be incited to bring in their positions and explicate underlying world views and value systems.

All discussions (lasting for 1.5 – 2 hours) were recorded and transcribed, the transcripts made anonymous and translated into English. The speaker codes used only provide information about gender (Mr./Ms.) and group membership (aff/lay: affected person/lay person); the country is also indicated: CYP: Cyprus, GER: Germany, NED: Netherlands, SWE: Sweden. The coding process (= assigning thematic categories to text passages) of the material was conducted by two researchers in parallel with Atlas.ti^® ^scientific software. The coding was compared and differences were adjusted. This approach reduced subjective bias. Since we were mainly interested in moral positions and cultural values, we followed a hermeneutic-analytical procedure common in social science based on a combination of qualitative content analysis [29] and Grounded Theory [30]: Interpretive concepts were applied to structure the material along our general research questions, but they were also developed inductively to identify main lines of argument. The eight FGs' transcripts were treated as one broad sample in which we compared inter-individual arguments to justify or reject specific positions. The final step of our analysis was the identification of main lines of argument by working out a qualitative typology of ideas and values lying behind them.

## Results

Although a full commercialization of organ procurement is rejected throughout all focus groups, the language of commodification, instrumentalization and exploitation seems to be densely interwoven with many argumentative threads of the discussion. Several participants refer to organ extraction as "harvesting" [Mr. N., NED_lay], "disemboweling" [Mr. M., GER_lay] or as taking out of "spare parts" [Ms. C., SWE_lay]. This imagery hints at the relevance which background conceptions of self and body already have for attitudes towards organ transplantation in general and not only for the commercialization of organ procurement in particular. Thus, the agricultural image of a field which is harvested might tend to elicit different answers to the question as to what should be permitted than the mechanistic imagery of the body as some piece of machinery with replaceable parts might suggest [31]. In the following section, the main types of images and conceptions of self and body which appeared in our focus groups will be described and explored with respect to their interdependence with attitudes towards organ donation as such and the commercialization of organ procurement in particular.

### "Just like cars": the body as private property

Many participants in all focus groups attach great importance to the idea of personal autonomy. For most of them, this idea also comprises bodily self-determination in the sense that one has the right to freely dispose of one's own body. In the context of organ transplantation, this right plays a crucial role when it comes to decision-making processes. Thus, a Dutch speaker would "start from the point that I have 100% self-determination over my own body" [Mr. N., NED_lay]. And a speaker from Cyprus declares: "What I will do with my body is my own business." [Mr. A., CYP_lay]

On closer examination, two different conceptions of bodily self-determination seem to present themselves. The first one is premised on the idea that no other person may make any claims with respect to one's own body or is allowed to interfere with one's decisions concerning it. In the group discussions, this "defensive" aspect of bodily self-determination is stressed when it comes to the question as to whether individuals have any responsibility or obligation to donate, be it towards the family or society at large. In these contexts, bodily self-determination is widely and vehemently postulated as "the right to refuse" [Ms. Q., NED_lay] donation, or, as these Swedish speakers put it: " [T]hey do not have the right to take my organs if I do not want them to." [Mr. B., SWE_aff] "No, it must happen by free will." [Ms. W., SWE_aff]

However, the mere absence of third parties' claims to my body or its parts does not necessarily imply that I myself am entitled to freely dispose of it as I wish. After all, there may still be limits to my bodily self-determination based on religious or "philosophical" considerations (see below). Thus, a German speaker who vehemently stresses the right to refuse donation also states: "I have problems with transplantations anyway because I believe ... that we can't prolong life artificially and just for kicks, or replace or manipulate it, because life as such ... has another sense than immortality" [Mr. U., GER_lay]. In this respect, the second conception of bodily self-determination goes much farther, the postulate being that one has an unrestricted right to actively do with one's body whatever one likes. In the focus groups, this "empowering" aspect of free choice and self-development in view of one's body primarily comes into play when future technological scenarios such as enhancement or infinite organ replacement are discussed and assessed:

" [...] as long as it remains your own choice – referring to what you said about getting new livers again and again while your mind wears away – as long as it remains your own choice whether you get a new liver or not then in my opinion there is no problem. When at a certain point you say: well, I am seventy years old, all this is not necessary for me, let me just await my own time, then isn't that just fine? [...] But if somebody else DOES choose to lengthen his life with new organs again and again, I think it is up to him." [Ms. R., NED_lay]

In the group discussions, the idea of bodily self-determination is frequently addressed in terms of ownership. The notion "that [...] my body belongs to me" [Mr. I., GER_lay] appears to be deeply rooted in everyday intuition because it is often presented as a consensual and nearly self-evident point requiring no further justification, as a Dutch speaker's argument against obligatory donation shows. As he says: "Everybody owns his own... has the right to dispose of his own body... It's my body." [Mr. N, NED_lay]

This idea of bodily self-determination in terms of ownership seems to bring the human body in line with other pieces of private property. On closer inspection, however, the application of this ownership paradigm does not necessarily imply approval of commercialization in the sense of making money with one's body or its parts. On the contrary, money often seems to be perceived as a factor which has the potential to impede self-determination by corrupting persons and distorting their own proper will, that way inducing them to do things they would not do otherwise. Thus, the autonomy and authenticity of decisions concerning the body can be called into doubt when financial motives are involved since this is seen as "something different than voluntary registration" [Ms. D., NED_aff]. Against the background of similar considerations, German participants discussing the obligatory psychological test in the case of living donation even compare financial incentives with other constraints on the freedom of decision such as psychological pressure in the family context.

On the other hand, arguments for bodily self-determination do show a certain affinity to a particular kind of body conception. Thus, especially when discussing the pros and cons of a future scenario in which self-preservation through infinite organ replacement becomes technically feasible, the participants frequently employ images from the sphere of handicraft or engineering which suggest analogies with the reparation of machinery in order to address and articulate their position: "It actually will be just like cars: Well, gosh, the radiator is broken or won't live long: out with it, put a new one in." [Mr. N., NED_lay]

Such descriptions of the human body within the framework of a mechanistic paradigm show a certain tendency towards accentuating aspects of functionality and performance when describing the body and its parts. These aspects are usually also described in terms of mechanistic and technological images such as automobiles, comparing organ transplantation to "fixing a car" [Ms. E., CYP_aff]. Such is the case in this statement made by a participant from the Netherlands:

"I have got 'Mercedes'-lungs. I had an argument with my physician: I want 'Mercedes'-lungs, or else I want to die. I mean it, I really said it like this. I have got 'Mercedes'-lungs, I don't want a 'Lada'. And then it was: Mrs. V. – I have got 'Mercedes'-lungs for you. Let's say it like this..." [Ms. V., NED_aff].

This mechanistic focus on functionality and performance seems to correspond to a tendency to relativize all other aspects of the body and thus to regard it like a mere "commodity". Hence, in discussing the provenance of a donor organ, some participants almost exclusively discuss the functional capabilities of the organ, explicitly denying that any other features (artificial/organic, human/non-human, living/dead, male/female) play any role. Moreover, from this perspective, organs are bereft of any symbolic meaning and do not have any significance for the person, the sentiment being that "only the mind can change somebody, the parts do not change a human being." [Ms. O., CYP_lay]

This disregard of all aspects except for functionality and performance certainly plays no small role in the fact that mechanistic body conceptions generally tend to promote quite a positive and optimistic attitude towards the technological possibilities of modern biomedicine, as is expressed in this statement from the Swedish group:

"So, one should probably not have an altogether negative attitude towards this [an overall replacement of organs]. Since it can easily become, as we said, like science fiction, let us replace this, let us replace ...that. Just like when you take your car to the garage, it is coughing and such, yes, let us replace that and fix that and then you are off again." [Mr. B., SWE_aff]

### "The human being is not a car": the body as part of a larger order

On the other hand, some lay people and patients in all four European countries also point out several limitations to autonomy and the free disposal of one's own body. One of these limitations arises from the belief that the body is not merely a piece of machinery with replaceable components, but an organic entity with its own intrinsic structure and dynamics which resist external interventions.

"But a human being is not a car." [Mr. F., NED_lay] "No. That's right. It's being loaded just as heavy, but it is not a car." [Mr. N., NED_lay] "And what do you mean by that?" [Moderator] "Well, a car: that is material, it doesn't talk back, though it does wear out too... but well, a human being is just something really different, a lot more sensitive too... well, how do you express that. It is not a thing, it is... well." [Mr. F., NED_lay]

In contrast to aforementioned mechanistic ideas, this more organicistic conception is rarely ever articulated in a direct and positive manner. This means that instead of explaining their conception of the body by means of explicit terms or images, the respective speakers often tend to address it indirectly, that is, by negating and rejecting mechanistic descriptions. Nevertheless, these conceptions of the body seem to have important implications for peoples' views and attitudes. They often correspond to a reluctant, skeptical stance towards science and the conviction that there are moral limits to technological possibilities. Especially when moral directives cannot be derived from the principle of self-determination, e.g. in the case of proxy decisions for deceased relatives, the conception of the holistic or organic nature of the human being and its body is brought to bear as a moral orientation which even has the potential to override relatives' presumptions:

"Well, if there is no decision, I mean it is clear that nothing should be taken out. I mean this is crystal clear. Because a human being as such is not a spare parts store. Well, for me, this wouldn't be ethically [Mr. M, GER_lay KNOCKS ON THE TABLE.] acceptable at all." [Mr. U., GER_lay]

This sense that certain limits are given can be related to the idea that nature itself has some sort of intrinsic, self-contained order that sets limits to all human interventions. In part, this notion is based on the religious image of nature as divine creation. Thus, under the premise that the human being was created by God and embodies divine will, one Dutch speaker interprets the body in a teleological manner as "a creation with a goal" [Mr. F., NED_lay].

On the other hand, the insistence on limits which is associated with organicist views is sometimes embedded in the non-religious idea of a natural order of things, which imposes certain limits on human action and invests human life with certain aims. This conviction often manifests itself when speakers refer to nature or natural entities like the body in a moral line of argument or qualify interventions in moral terms, calling them "natural" or "unnatural."

Such underlying notions of a natural order are sometimes accompanied by the conviction that one has the responsibility to leave the body as it is and instead adapt one's own behavior or way of life to the conditions set by one's natural bodily constitution. Thus, the aforementioned German speaker continues, saying that "I have no right to change my body in a way that I change parts there. Well, if it no longer functions, I will have to find a way to deal with the consequences of that, that is to say to deal with it without operation" [Mr. U., GER_lay]. And a participant from the Dutch group seems to proceed from the idea of a divine natural teleology in which everything that occurs has a function:

"I think you have been created for good reason, with two kidneys." [Mr. F., NED_lay] "You have a kind of back-up inside." [Mr. N., NED_lay] "Yes." [Mr. F., NED_lay] "You shouldn't start fiddling with people in this respect. See, those things are there, you are born like that and they have a function. Why do you have two, why not one?" [Mr. Y., NED_lay]

In this line of thought, the body is often perceived less as a passive, irresponsive object which is separate from the self than as something more or less congruent with the person as such. One way to conceptualize this monistic intuition of an embodied self is to speak of the body as an instance with its own inherent "authority" or "wisdom" which can influence a person's attitudes, behaviors and lifestyles:

"I guess there are also signs somehow from your body, that, in a way you can't continue your life-style or something like that ... No idea, if you smoke. Someday you'll get, everything doesn't look like it should be anymore or so. I think all these things are hints and challenges, which will help you in a way to find out what to change, and that you can change it, and that it needs to be done." [Ms. G., GER_lay]

Such monistic conceptions can have a great impact when it comes to attitudes towards organ donation. Thus, they seem to promote the idea that (a part of) the donor lives on in the body of the recipient via organ transplantation. As this affected speaker from Cyprus explains, if body and soul are – more or less – congruent, then a transfer of physical parts can appear as a transfer of portions of one person to another:

"I would know that a part of my child would breathe and live in another body and I would have a part of my child in life. Apart from saving a life, a part of child, either an eye or a kidney or whatever, would be alive." [Ms. E., CYP_aff]

The notion that (a part of) the donor lives on in the recipient via organ transplantation makes it difficult to view or treat organs as fungible commodities. Thus, many speakers speculate as to whether attributes of the donor "may be transferred to somebody psychosomatically and change a person." [Mr. H., CYP_aff] Although such considerations are often articulated in a slightly facetious manner, they still play a major role in discussions, especially for the affected people. Attributes which are considered to be potentially transferable are e.g. character traits, preferences or aversions, talents or even hobbies:

"I would like to say something I read in a newspaper seven years ago in Canada. A woman received a kidney from a deceased donor. When she went home she wanted to have a beer and a hamburger everyday at lunch time, something that she never did in her life. Thus, she wanted to find out who the donor was. She discovered and the donor used to do this everyday at lunch time..." [Mr. K., CYP_aff] "So, these things are transferred." [Mr. H., CYP_aff]

### "The brain makes us special": meaningful organs

The aforementioned complexes of self and body conceptions do not constitute two distinct, monolithic blocks. There are contradictions within the camps and floating boundaries between them. Thus, within the dualistic framework of the property paradigm, the brain often receives a specific status. It is identified as the physical basis of a person's mind, its anchorage in the body, so to speak, and thus the locus of personal identity. In this perspective, the brain consequently marks a logical and technical limit of bodily self-determination since it accommodates the self itself: the subject of self-determination.

"There is one single organ that of course cannot be replaced – that's the brain. That's what makes us special. If we did replace it, in reality we actually wouldn't replace the brain, rather we would give the brain a different body. Since it is the brain that makes you a personality. Other than that I'm in favor of replacing all organs as soon as this can be done technically, biologically." [Mr. S., GER_aff]

Interestingly, one Dutch participant also holds that the genitals should be excepted from transplantation because he regards them as relevant for personal identity, as well, due to progenitory considerations:

"I think there are two organs that certainly shouldn't be transplanted and those are the brains and the genitals. Because I think they do influence who you are. Brains definitely, I think that you become a wholly different person. Namely the other person. They can not do it [now], but if they could. And with the genitals – you do not beget your own children, but someone else's, suppose you would undergo a transplant as a man. So that's ... not even allowed." [Mr. T., NED_aff]

Eventually, in one Cypriot group, a discussion about the status of the eyes evolved, indicating that some participants also consider them as relevant to personal identity. In this context, the special status of the eyes is not based on their visibility, alone; they are rather described as a kind of gateway to the person's inner self:

"Ms. X. has said something earlier. That is, "I think it is better if the eyes of the child live..."" [Moderator] „Not only the eyes. But the issue of eyes has impressed me because I had heard about such a case in the past.” [Ms. X., CYP_lay] "Would it be remarkable because we see the eyes whereas we don't see the liver or the kidney?" [Moderator] "You see the person through the eyes" [Ms. O., CYP_lay].

The view that the brain has an exceptional status has significant consequences for an evaluation of organ transplantation in at least two respects. First of all, it establishes certain reasons for accepting the occurrence of brain death as marking the death of a person as such. If personal identity is an exclusively spiritual phenomenon which is based on brain functions but detached from the rest of the body, an irreversible breakdown of brain functions simultaneously marks the death of the person as a whole, leaving only a "living body":

"I think that the body may be still alive but to me this person would be dead.... perhaps I may not say that he/she is dead, perhaps I would say that the body lives and therefore I would kill it by myself in order to take the organs and give them to somebody else... because the body lives. But in this case I would perhaps say that "yes, I have no problem" because I would give life to other people. Perhaps I would take life by myself but I would give it to other people because I would know that it was over." [Mr. L., CYP_lay]

Secondly, on the basis of the notion that the brain has a particular status, several speakers, especially in the groups of lay people, also strictly reject any speculations about the possibility of transmitting personality or personal characteristics through transplantation of other organs, since " [p]ersonality does not come with the heart." [Ms. P., SWE_lay]:

"But it is a technical organ, I mean, someone's hobbies don't reside in his kidney. Perhaps it resides in your brains, but my kidneys don't indicate that I love sport. At the utmost they have been influenced by that, that perhaps they are in better shape, but that implies only their technical state. And that could perfectly fit in someone else's body." [Ms. Z., NED_lay]

A transplant receiver from the Dutch group who declares that he is „not so sensitive to what I will disrespectfully call "ghost stories"” (i.e. personality-transmission narratives) even offers an alternative, exclusively naturalistic explanation referring to

"...the physical phenomenon that a tissue has a certain dependence on a certain substance, you know your 'tostis', and that the desire comes with that. ..." [Mr. J., NED_aff]

Interestingly, in the course of the discussion, the selfsame patient also states that his down-to-earth naturalism might be a coping strategy to avoid emotional stress. This stress could be induced by thinking about "his" organ donor and "to protect myself from that, also because I'm afraid I would get too carried away" [Mr. J., NED_aff].

## Discussion

Throughout all group discussions, we actually found a great number of references to cultural images and conceptions of self and body. On our level of analysis we could not detect any national differences. Three main positions seem to present themselves in all four European countries: On the one hand, arguments in favor of maximum bodily self-determination are often articulated in terms of ownership of the body. In this context, body conceptions which address the body as some kind of machine composed of single elements which can be replaced by functional equivalents are particularly prominent. In contrast, limits to bodily self-determination are often expressed against the background of a more or less articulate notion that human beings and the human body belong to some higher realm, be it that of divine creation or of a natural order of things. Finally, there are arguments which accentuate specific organs, mainly the brain, but also the genitals and the eyes, thus implying more differentiated conceptions of bodily self-determination and unavailability.

These findings show certain similarities to the results of previous studies [32]. However, the body-self conceptions we found apparently do not possess the character of explicit positions based on articulate arguments; on the contrary, they seem to operate as background notions that are deeply rooted in particular cultural customs and traditions [33]. Moreover, their connections to peoples' attitudes towards organ donation and its commercialization are far more complex and difficult to trace than the scheme of Joralemon and Cox suggests [25]. Thus, although the participants frequently refer to the notion of ownership when talking about the human body, this does not necessarily imply that they consider the body as some piece of private property available for commerce. On the contrary, the concept of ownership often rather seems to serve as a metaphor for autonomy and bodily self-determination, principles which can as well imply a rejection of commercialization. In this respect, the idea of self-ownership seems to be at least ambivalent [34]. Moreover, claiming one's right to bodily self-determination can serve as a basis for rejecting third parties' claims to one's body and organs as well as for justifying extensive use of the technological possibilities of transplantation medicine. Those who held that limits are placed on bodily self-determination, on the other hand, often based their arguments on convictions which refer to natural order or divine creation. These conceptions, however, can be used to reject organ transplantation or commodification as unnatural or against god's will, but also prepare the ground for the idea that a person has no exclusive rights over her own body. Finally, there is evidence of certain "organocentric" conceptions of the body which identify particular organs as central to personal identity. Such organs may demarcate a definite limit to any technical modification, transmission or commercialization, the notion being that these practices would affect the personhood and self-understanding of both, donors and receivers. But their identification seems to depend on socio-cultural and bio-philosophical background assumptions which are embedded in culturally and historically variable contexts (see table 1).

## Conclusion

Although more and broader representative research on these topics would be necessary and valuable, our sample allows conclusions that could inform applied ethics and constitute hypotheses for further sociological and anthropological research. Thus, the way we found lay people and patients to think about the body and organ donation may correspond with some of the academic philosophical and anthropological positions on commodification described above. But in contrast to academic authors' restriction to (or preference for) one single paradigm, we observe a plurality of different body conceptions among the public which are interwoven with their attitudes towards transplantation medicine in rather complex ways.

Against this backdrop, we conclude that the language of commodification is much too simple to capture what is at stake in everyday life intuitions about organ donation and organ sale. Those who base their evaluation of commercialization in organ procurement on a (pro or con) stance to commodification should be aware of the variety of ideas regarding the human body. This is necessary in order to reflect the anthropological assumptions implicit in their own arguments and their prevalence on a socio-political level (for the public and those who would be motivated to donate or sell their organs). Whether there is any intersubjectively graspable way of ever proving the adequacy or plausibility of a particular conception of the human body is doubtful, however. Therefore, the more interesting question is how bioethical discourse and political practices confront the plurality of body-self conceptions in the public as a given reality. We see at least two basic possibilities:

On the one hand, one could defend an "anthropologically informed" position, arguing that the body has a particular constitution which makes it either resist or suit commodification. This position would take on a certain expertocratic air since it would seem to claim definitive objective insights into the nature of human beings and their corporeal existence. Besides the epistemological question of how such insights could be gained and justified in the first place, it would be interesting to see how this approach would handle or "sublate" recent plurality. How, for example, would it deal with uninformed, ignorant or disinterested lay people who simply insist on having their own views on body and self? Given the fact that in modern, liberal democracies not superior insights, but the will of the majority determines (within the limits of constitutional rights and democratic procedures) the legitimacy of political decisions, there seems to be no way of imposing certain body conceptions on those who simply do not accept them. Thus, when it comes to ethical recommendations and political consultation, anthropological approaches will have to find a way to incorporate public attitudes and psychological images as well as socio-cultural conceptions of the body instead of simply insisting on the superiority of particular expert theories.

On the other hand, one could take a liberal approach and try to get rid of all substantial anthropological or metaphysical assumptions from the very start in order to approximate a "neutral" framework for the peaceful coexistence of a plurality of worldviews. In this spirit of tolerance, however, one must be all the more aware of some liberal thinkers' affinity to Lockean self-ownership and the distinct world view of modern science which imply and promote particular mechanistic conceptions of nature, the self and the body [23]. To this end, a liberal position might have to abandon the pretence that they take an agnostic perspective on the body and refrain from addressing existential questions, engaging – instead – in an explicit discussion on the plurality of existing conceptions of the body. This way, liberality would not demand exclusion of the body from public discourse, but rather explicit admission, acknowledgement and protection of the plurality of ideas which are attached to it, e.g. through the development of legal regulations which are not based on any fixed body-related conceptions.

The sketched heuristic distinction between the "anthropologically informed" approach and the liberal approach should not be confused with other prominent classifications such as the one of "bioconservatism" versus "transhumanism" in the debate on human enhancement [35]. Our distinction is located on a more general level insofar as it refers to bioethical and political approaches towards the plurality of body conceptions while "bioconservatism" and "transhumanism" rather stand for two specific positions *within *this plurality. Hence, in the perspective of our distinction, both positions can be advanced in an "anthropologically informed" as well as in a liberal manner, depending on their way of dealing with plurality. Thus, the "transhumanist" stance that "current human nature is improvable through the use of applied science and other rational methods" ([35], p. 202) rather seems to express an "anthropologically informed" approach because it obviously claims insights into the existence and qualities of human nature. And the "bioconservative" counter position can definitely represent a liberal approach as long as it does not rely on a particular conception of human nature, but rather mirrors respect for actual "bioconservative" consensus among the general public.

Another prominent bioethical and political debate in which the capacities of both, „anthropologically informed“ approaches as well as liberal approaches, are challenged, evolves around the problem of death in the context of modern biomedicine. On the one hand, a conception of death, explicit or not, is of central importance for many fields of biomedical practice, from organ procurement policies to the withdrawal of life sustaining treatment. On the other hand, however, it is widely agreed that death is not simply an objective scientific fact that can be determined by means of empirical research. Understandings of death are always embedded in cultural, religious or metaphysical views. Nowadays, there actually exists a wide plurality of such world views which can lead to quite different conceptions of death [36]. In this situation, the State of New Jersey's law on brain death [37], as well as more recent legislation in Japan [38] seem to point towards a liberal strategy which allows the individual to choose a definition of death for him or herself. From an anthropologically informed point of view, however, it can be questioned whether this really constitutes a satisfactory solution. After all, assuming (and be it just for the sake of the argument) that there is one definite answer to the question of death, a contravening practice would simply amount to an instance of killing, although perhaps at the request of the person killed.

Against this background, the two approaches could possibly rather be understood in the sense of two diametrical, but at the same time complementary and mutually corrective perspectives. Both seem to be needed for an adequate consideration of plurality in bioethical and political debates touching upon body conceptions: The liberal perspective, on the one hand, makes us aware of the wide range of individual ideas regarding the body in modern pluralistic societies, thus preventing us from naïvely taking our own intuitive views as self evident facts. And the "anthropologically informed" perspective, on the other hand, provokes us to really take each of these conceptions seriously, respect their claims for validity and consider their ideational and normative implications as well as their social consequences – rather than just content ourselves with comfortable illusions of indifferent coexistence or a superficial "anything goes".

## Competing interests

The authors declare that they have no competing interests.

## Authors' contributions

Both authors have contributed equally to the article.

## About the authors

MS (M.A.) is research associate at the Department of Medical Ethics and History of Medicine at the University Medical Center Göttingen, Germany. His research is concerned with biomedicine and bioethics in their socio-cultural contexts as well as with questions in ethics, political philosophy, and the history of philosophy.

SS, Doctor of Science, is Professor for History, Theory, Ethics of Medicine in the Department of Medical Ethics and History of Medicine at the University Medical Center Göttingen, Germany. Her main research interest is the mutual relationship between cultural and ethical aspects of biomedicine as well as the role of body concepts for bioethical reflection.
